# Electrostimulation of the lingual nerve by an intraoral device may lead to salivary gland regeneration: A case series study

**DOI:** 10.4317/medoral.22597

**Published:** 2018-09-28

**Authors:** Andy Wolff, Meltem Koray, Giuseppina Campisi, Frank P. Strietzel, Gloria I. Lafaurie, Ben Z. Beiski, Jörgen Ekström

**Affiliations:** 1 Saliwell Ltd., Harutzim, Israel; 2 Oral Surgery and Medicine, Department of Oral and Maxillofacial Surgery, Faculty of Dentistry, Istanbul University, Istanbul, Turkey; 3 Oral Medicine, Dipartimento di Discipline Chirurgiche Oncologiche e Stomatologiche, Università di Palermo, Palermo, Italy; 4 Charité Center 3 for Dental, Oral and Maxillary Medicine, Department of Oral Medicine, Dental Radiology and Oral Surgery, Charité - Universitätsmedizin Berlin, Berlin, Germany; 5 Unit of Basic Oral Investigations-UIBO, School of Dentistry, El Bosque University, Bogotá, Colombia; 6 Department of Pharmacology, Institute of Neuroscience and Physiology, The Sahlgrenska Academy at the University of Gothenburg, Gothenburg, Sweden

## Abstract

**Background:**

Salivary gland function is controlled by the salivary reflex, whose efferent arm is composed by the parasympathetic and the sympathetic divisions of the autonomic nervous system. Parenchymal injury is the main salivary gland involvement of Sjögren’s syndrome and head and neck radiotherapy, but neural damage has been reported as well. Recently an intraoral device for electrostimulation of the lingual nerve in vicinity to the lower third molar has been introduced. At this point this nerve carries efferent fibers for the innervation of the submandibular, sublingual and several minor salivary glands and afferent fibers of the salivary reflex. Therefore, excitation of these fibers potentially leads to increased secretion of all salivary glands. Thus, the study objective was to assess whether comprehensive neural activation by electrostimulation of the lingual nerve carries the potential to induce the regeneration of damaged salivary glands.

**Material and Methods:**

The device was tested on three patients with no collectable resting and stimulated secretion of saliva during a double blind, sham controlled period of two months and nine open-label months.

**Results:**

All three subjects developed the capacity to spit saliva, not only in direct response to the electrostimulation but also after free intervals without electrostimulation. In addition, their symptoms of dry mouth severity and frequency improved.

**Conclusions:**

This recovery is probably due to the combined effect of increase in secretory functional gland mass and regain of nervous control of the secretory elements and blood vessels. Both are phenomena that would contribute to gland regeneration.

** Key words:**Xerostomia, dry mouth, saliva, electrostimulation, regeneration.

## Background

Saliva is of outmost importance for oral health and quality of life. Lack of saliva causes dry mouth, weakened taste acuity, dental caries and candidiasis, and difficulties in speech, mastication and swallowing (1). Sjögren´s syndrome and head and neck radiotherapy are characterized by salivary gland parenchymal destruction and hypofunction, but neural damage has been reported as well (2,3). Though parasympathomimetics (pilocarpine, cevimeline) are effective sialogogues, their use is hampered by adverse effects such as sweating, diarrhea, urinary urges, bronchoconstriction and hypotension. Moreover, due to the co-morbidities in the elderly, their use is frequently contraindicated (4).

**Figure 1:**
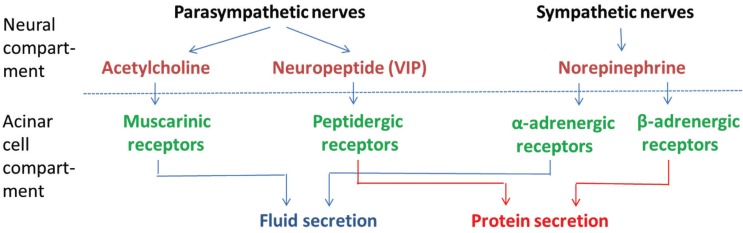
Schematic overview of the neural control of salivary gland function. Acinar cells (below) are prompted to produce saliva by neurotransmitters (above) that bind specifically to surface receptors. The parasympathetic arm releases acetylcholine and neuropeptides (i.e. vasoactive intestinal peptide, VIP) that bind to muscarinic (M1 and M3) and peptidergic receptors, respectively. The sympathetic neurotransmitter norepinephrine binds to α1- and β1-adrenergic receptors. Fluid secretion is mainly the result of muscarinic and α1-adrenergic activation, whereas protein production derives mainly from the stimulation of peptidergic and β1-adrenergic receptors.

**Figure 2:**
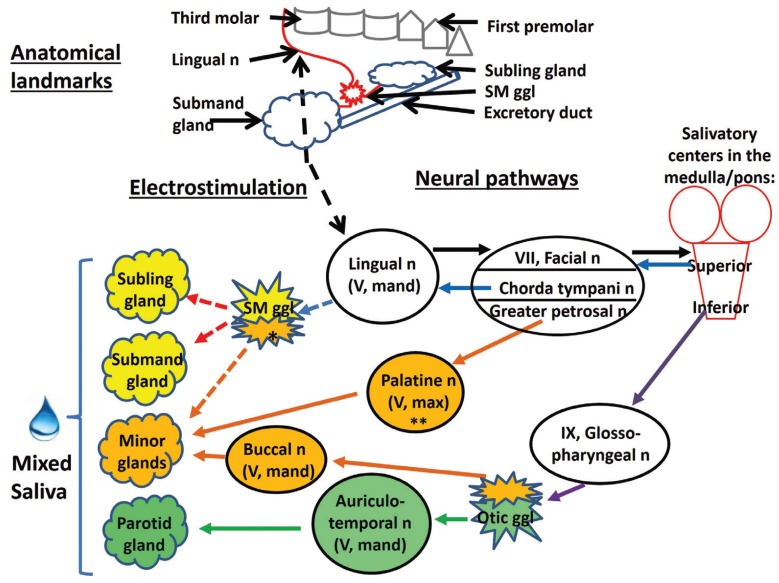
The upper part (“Anatomical landmarks”) shows the location of the electrostimulation in relation to relevant anatomic structures: third molar, lingual nerve, submandibular ganglion and gland, sublingual gland and the excretory duct. It should be noted that the figure does not depict neither fibers that detach from the submandibular ganglion in direction to minor salivary glands nor the sensorial origins of the lingual nerve. The lower part (“Neural pathways”) provides an overview of the consequences of the lingual nerve electrostimulation (black dashed arrow) on the salivary reflex at the parasympathetic arm. The black full arrows represent afferent activity, while all other arrows denote efferent activity, as follows: - light blue arrows: fibers going up to the submandibular ganglion (SM ggl), - purple arrows: fibers going to the otic ganglion (Otic ggl), - red arrows: fibers originating from the submandibular ganglion and innervating the submandibular (Submand) and sublingual (Subling) glands, - orange arrows: fibers to the minor salivary glands (Minor glands), and - green arrows: fibers to the parotid gland. Dashed light blue, red and orange arrows denote fibers that carry impulses derived from both, direct and reflex stimulation. Other abbreviations: n (nerve), mand (mandibular branch), max (maxillary branch), ch (chorda), ggl (ganglion). The single asterisk indicates that the lingual nerve contributes to minor gland innervation also via Remak´s intralingual ganglia in addition to the submandibular ganglion, while the double asterisk denotes that the palatine nerve originates from the sphenopalatine ganglion (12). Note that sympathetic nerves can be expected to act on the glands as well, as an effect of the stimulation of the reflex arc. Reflexly elicited sympathetic impulses, originating from the upper thoracic paravertebral sympathetic trunk, reach their targets via sympathetic nerve fibers following the arteries of the glands; the relay between pre- and postganglionic sympathetic fibers is the superior cervical ganglion. However, the minor glands are thought to lack a sympathetic innervation of their acinar cells (12).

Salivary glandular functions are regulated through reflexes elicited by alimentary activities. The parasympathetic innervation causes the main part of the salivary flow, but notably parasympathetic and sympathetic innervations interact synergistically by various acinar receptors (Fig. 1) (5). Furthermore, nerves exert long-term trophic effects, as illustrated in animal experiments: gland size and secretory capacity adapt to prolonged variations in the intensity of the reflex stimulation (6). In a recent clinical trial, consisting of a crossover sham-controlled phase followed by an open-label phase (7,8), electrostimulation of the lingual nerve on one side by an intraoral device applied regularly in brief periods, was shown as a safe and efficacious way of decreasing oral dryness in a study population of about 100 patients with xerostomia of various etiologies. Electrostimulation aimed at activating not only efferent (preganglionic parasympathetic) nerve fibers of the ipsilateral submandibular and the sublingual glands but also afferent fibers resulting in global (bilateral) salivary reflex responses (Fig. 2).

Three patients, a sample of the study population, that had no collectable saliva initially, increased their secretory capacity over 11 months, at the regular use of the device.

The clinical results of these non-secreting patients are reported, each with a different diagnostic background - primary and secondary Sjögren´s syndrome, and head and neck radiotherapy.

## Material and Methods

- Device description

**Figure 3:**
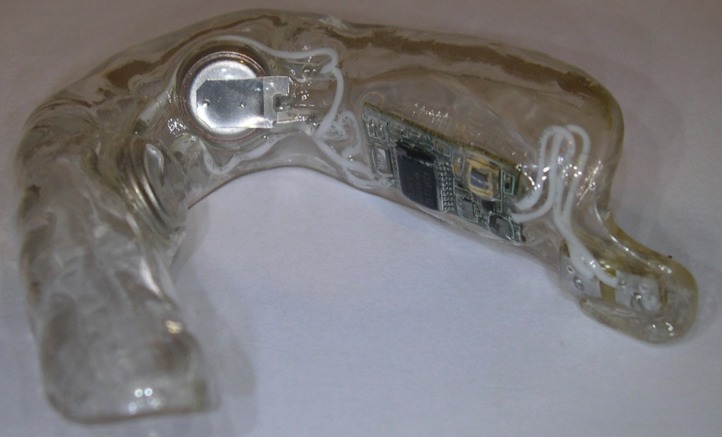
The GenNarino device with the rectangular electronic circuit on its right side and the round battery on the anterior part. A pair of stimulating electrodes protrude from the buccal surface on the extreme right side extension (not shown in this lingual view-picture). The latter are positioned in such a manner that they are in contact with the oral mucosa in the mandibular third molar area, in the immediate proximity to the lingual nerve. As the flange is separated from the mucosal sur-face, the electrodes don’t prick the tissue.

GenNarino™ is an individualized mouthpiece containing an electronic circuit with a microprocessor, a pair of stimulating electrodes and two 3V 30mAmp/h batteries (Fig. 3). The electrodes contact the oral mucosa in the mandibular third molar area, close to the lingual nerve on one side (Fig. 2). The electrical stimulation is of low intensity and not felt by the patient. This feature enables an ideal scenario for undertaking double-blind sham-controlled studies (9). In this trial the stimulation signals were pulse-trains at 5Hz, biphasic, at rectangular pulses of 1mSec, with an output of 150µA.

The patient activates and deactivates the electrical stimulation by pressing the “ON” and “OFF” buttons of the remote control, respectively. An amber light on GenNarino that blinks upon activation of the remote control ensures that the device is working as intended by design. Failure to blink means that the device is not functional and needs to be returned, and that a new one has to be ordered.

- Study design

The study was conducted in full accordance with ethical principles, including the World Medical Association Declaration of Helsinki and the additional requirements of the countries where the research has been carried out. The study was approved by all ethical boards and registered at ClinicalTrials.gov (US National Institutes of Health, Identifier: NCT00509808). All study subjects gave their informed written consent.

Xerostomia patients were recruited in 14 institutions of 13 countries. The overall results of this trial have already been published (7,8). This paper reports the assessments obtained from all the subjects that started the study with a salivary flow rate judged as zero and showed up to all the follow-up sessions until the 11-month study completion (n=3).. They were evaluated at Charité Universitätsmedizin Berlin (Germany), İstanbul Üniversitesi (Turkey), and Università di Palermo (Italy).

This prospective trial was divided in 2 stages. The first stage (Stage I) was randomized, sham-controlled, crossover and aimed to determine if electrostimulation has an additive effect on mechanical stimulation achieved by GenNarino’s foreign body effect in the mouth. The second stage (Stage II) was open-labeled and aimed to assess long-term influence of GenNarino on xerostomia parameters. During both stages subjects were instructed to use the device not more than once every hour but otherwise as many times as they liked every day.

In Stage I, GenNarino was used for 10 minutes at a time in either “sham” (mechanical stimulation) or “active” (mechanical and electrical stimulation) mode, each during one month in a double blind fashion. This time period was chosen since it was used in a preliminary proof of the principle study, in which the device was used in a clinic during 10 minutes (9). The sequence of “sham” and “active” use was assigned randomly to each patient. Identically looking remote controls were assigned to each subject, with pre-coded software commands set for either not activating (“sham”) or activating (“active”) the electrical stimulation upon pressing the “ON” button. Both, patients and investigators, were blinded to the type of stimulation (mechanical only or mechanical-electrical) schedule. Stage II was open-label where only “active” devices were used after completion of Stage I for 9 months to assess the cumulative effect of electrostimulation from baseline, throughout the “active” month of Stage I until the end of the study. At the beginning of the study, together with the Stage I randomization, subjects were also randomly allocated to use the device during each trimester of Stage II either 1, 5 or 10 minutes at a time.

- Outcome measures

The devices were manufactured by the study initiating company, Saliwell Ltd (Harutzim, Israel) using impressions taken from the subjects’ dental arches. After baseline, outcome assessments took place at the end of the 1st, 2nd, 5th, 8th and 11th month of device use. At each follow-up, questionnaires, whole saliva and safety-related information were collected. The primary outcome (xerostomia severity) and patient-centered secondary outcomes were measured by a previously validated questionnaire (10). This paper reports the assessments most directly related to salivary gland function, i.e. resting and stimulated salivary flow rates and the replies to the questions “How dry is your mouth today?” (“dry severe”) and “How often does your mouth feel dry?” (“dry frequent”). Answers to the question “dry severe” were reported using 10 cm long Visual Analogue Scales running from the worst condition on the left to the best on the right end of the line, and to the question “dry frequent”, with possible responses: Always / Frequently / Occasionally / Never, rated 1/2/3/4, respectively).

Resting and stimulated salivary flow rates were assessed always at morning hours. Patients were requested to take nothing into their mouth for 90 minutes or longer, and then to spit during five minutes into containers (F.L. Medical, Padova, Italy, catalogue #25174), while avoiding swallowing. The containers were closed immediately after collection to avoid fluid evaporation. Salivary flow was stimulated by chewing a piece of parafilm. Saliva volume was determined gravimetrically (assuming a specific gravity of 1.0) (11).

As safety-related secondary outcome measures, vital signs (blood pressure and heart rate), changes in health condition (as reported by the patients) and oral mucosal status were assessed.

## Results

- Subject BG

This 52 year old, Caucasian female suffered from mixed collagenosis and myalgia. Serologic laboratory parameters were found positive for anti-nuclear antibodies and enhanced extracted nuclear antigens. Despite no histologic findings of inflammatory foci in a biopsy of minor salivary glands from the lower lip submucosal tissue, secondary Sjögren’s Syndrome was considered to be her diagnosis. During the five years preceding the present study, the patient developed symptoms of oral dryness, severely interfering with her quality of life.

At time of admission, the regular medication comprised oral intake of pilocarpine (5 mg three times per day) which was ceased prior to inclusion into the clinical study. Apart from that, the patient took the immunosuppressive drug azathioprine 75mg twice daily and thyroxine 150µg per day regularly. On month 4 of the study, estrogen replacement was prescribed additionally.

During the study progress, the frequency of using the device was reduced from 6 to 5 times daily between visits 2 and 3 (corresponding to the blinded active or sham device usage period) as well as from 5 to 4 times daily between visits 3 and 4. From visit 4 onwards, the frequency of using the device was further reduced from 4 to 3 times daily. Nevertheless, prior to visit 4 the patient reported frequent subjective feeling of oral dryness, whereas she reported occasional feeling of oral dryness afterwards, despite the reduced frequency of using the device.

- Subject IT

This 62 years old male patient was referred to the Oncology Department due to neck swelling. Further to a biopsy from nasopharyngeal tissue he was diagnosed with undifferentiated nasopharyngeal carcinoma (type 3). He received chemotherapy with three doses of 200mg cisplatin in 2004. In 2005 he was treated with radiotherapy at the nasopharyngeal area at a dose of 70Gy in 28 fractions, and bilaterally to the neck area at a dose of 50Gy in 28 fractions. He had severe xerostomia during the radiotherapy and thereafter.

He used the device 10-15 times daily throughout the study and was not medicated to treat xerostomia. When asking to add comments in addition to the structured questionnaire he declared that the device is ineffective after the one sham month, and the he “cannot live without the device” at the end of the study. Up today, there was no cancer recurrence and his stimulated whole salivary flow rate is normal with a value of 1 ml/min.

- Subject PI

This 69 years old female patient had a definitive diagnosis of primary Sjögren’s Syndrome, based upon minor salivary gland biopsy and serological tests. As the disease affected severely her quality of life, the patient took regularly, in addition to xerogenic and antihypertensive drugs, also antidepressant medication.

At time of admission, the regular medication for Sjögren’s syndrome comprised oral intake of pilocarpine hydroxychloroquine and topical sialogogues. However neither pilocarpine nor any other systemic sialogogue were used during the trial.

During the study period, the frequency of using the device increased from 2 to 3 times daily between visits 4 and 5. Subjective feeling of difficulties in swallowing and speaking slightly decreased during the study period, whereas she reported constantly feeling of oral dryness, despite changes of frequency in using the device.

Summary of findings

 Table 1 summarizes the findings of the three subjects. In general, the pattern of the oral dryness symptoms was: 1) severity of xerostomia seemed to improve from baseline to month 11 for subjects BJ and IT and to remain stable for subject PI; 2) frequency of xerostomia appeared to improve from baseline to month 11 for subjects IT and PI and remain stable for subject BG; 3) for subjects BG and IT the month “active” had better outcomes, in comparison to the month “sham”, in regards to of dryness severity but was similar for all subjects in terms of dryness frequency; 4) the trends of subjective parameters were roughly correlated with the objective ones.

**Table 1:**
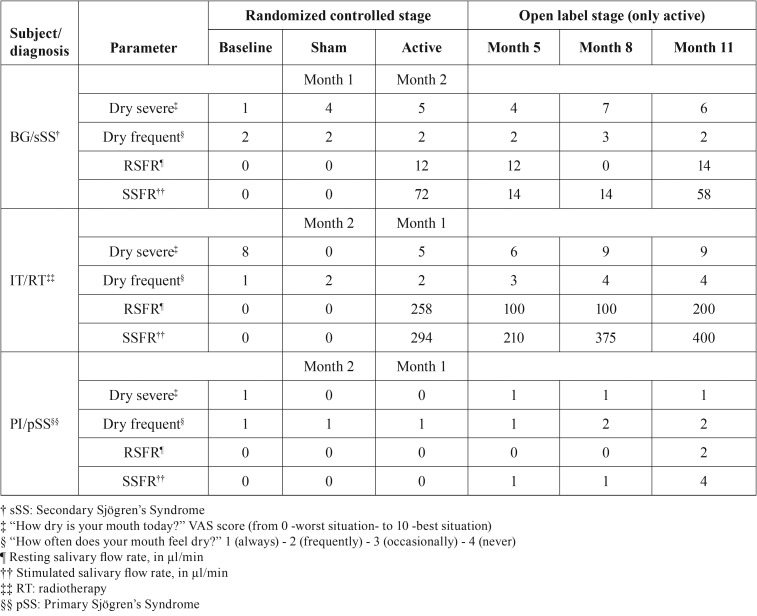
Summary of the findings of the three subjects.

Salivary flow rates of all 3 patients, either resting or stimulated by chewing, were 0 at baseline and after one month of “sham” device use, whether this month was the first follow-up month (subject BJ) or it followed the month of “active” device use. Among patients BG and IT, resting and stimulated saliva could be collected after the first month of “active” device use, whether this month was the first follow-up month (subject IT) or it followed the month of “sham” device use. In subject PI a small volume of stimulated saliva could be collected at the end of month 5, but unstimulated saliva was only collectable at the end of the trial. For all 3 patients, once the first time saliva could be collected, stimulated flow rate never returned to 0 after using the “active” device. In regards to resting saliva, except for the month 8 collection of subject BG, it was always collectable after its first appearance upon “active” device utilization.

For all three subjects, no significant changes in the vital signs and in the oral mucosal status were detected.

## Discussion

While unilateral electrostimulation of the oral mucosa on the lingual side of the third molar region was accompanied by increases in salivary output and relief of xerostomia in the three patients, other consequences related to lingual nerve activation such as pain and taste sensations were not experienced. Thus, through the excitation of the lingual nerve, the device is likely to (a) trigger the afferent large Aß fibers, which relay modalities of touch-pressure, vibration, and possibly proprioception and, thus, to evoke the salivary reflex engaging different types of salivary glands bilaterally, and to (b) stimulate directly the efferent secretomotor parasympathetic fibers to the ipsilateral submandibular and sublingual glands (12). The device is, however, unlikely (c) to stimulate the small somatic Aδ and C fibers for pain and temperature sensation as the output of the device (150µA) is well below the pain tolerance threshold in the alveolar ridge (1500µA), and (d) to stimulate the special afferent (taste) components as opposed to the taste response that can be evoked by the application of electrogustometry on the tongue taste buds (13).

Previous studies using electrostimulation have either aimed at activating only afferent nerves of the glands, by a hand-held device placed between the tongue and the palate (14), or at activating only the efferent nerve of the parotid gland (i.e., the auriculo-temporal nerve), by transcutaneous nerve stimulation (TENS) applied to the parotid region (15). Thus, direct and simultaneous stimulation of both afferent and efferent nerve fibers of the salivary reflex did not occur. A further disadvantage in those studies was the big size of the devices and the evidently unwieldy use of them.

A shortcoming of the present study is its small number of subjects. Another significant drawback of the long term stage (months 3-11) of the current study is the lack of comparison with a sham device serving as control. Notwithstanding, sham devices exert a mechanical stimulation. It is also well known that without treatment, salivary gland function and the perception of oral wetness are not expected to improve or may even worsen within the time frame studied here (16,17).

Although salivary gland hypofunction is not an absolute indicator of the subjective symptoms of xerostomia, the patients presently under study shared a pattern of parallelism between functional (measured by flow rate) and symptomatic (assessed by questionnaire) recovery. However, their strength of response was dissimilar, with recovery of subject IT being the strongest and that of subject PI very poor. Scrutiny of the findings raises a series of questions:

1) What is the reason that patient IT had an impressive subjective and objective recovery, despite the massive damage to the salivary glands as a result of the high-dosage radiotherapy delivered to treat his malignancy? Radiotherapy for nasopharyngeal carcinoma encompasses all the salivary glands, damages all gland structures and causes the most severe gland hypofunction and xerostomia (17) and further, regenerative therapy of this pathology is an arduous challenge (18). This particular patient was extremely compliant with the study protocol. He used the GenNarino device 10-15 times per day throughout the entire study length. Perhaps this intensive and repeated electrostimulation potentiated the positive outcome.

2) Why the recovery of subject BG, suffering from secondary Sjögren’s Syndrome, was much better than for subject PI, suffering from primary Sjögren’s Syndrome? As the frequency of device usage was similar for both patients, it might be interesting to assess an impact of the type of underlying disease. In fact, a study found a higher frequency of oral symptoms and stronger B cell activity (autoantibody production and lymphocyte infiltration) in primary Sjögren’s Syndrome compared to secondary Sjögren’s Syndrome (19).

The probable cause of the reappearance of collectable salivary output in the three patients is the re-establishment of functional neuro-effector junctions accompanied with regeneration of gland tissue as judged by preclinical studies. In general parasympathetic innervation is required for salivary organogenesis by maintenance of the epithelial progenitor cell population (20). Long-term studies on adult animals show the importance of the parasympathetic nerves in the recovery of salivary glands subjected to atrophic influences: (a) upon parasympathetic denervation and allowing time for re-innervation, the atrophic glands gain in weight and the reflexly elicited secretory response returns (21,22) and (b) upon extreme atrophy, such as after a transient period of duct-ligation, while providing for the maintenance of the parasympathetic innervation, salivary glands retain the ability to regenerate (23).

By electrical stimulation of the parasympathetic innervation or by reflexly elicited parasympathetic nerve activity through mastication, the mitotic activity of the glands increases (24-27). Though, the sympathetic innervation seems of minor importance for the gland size, as judged by denervation experiments (28), there are reports of increases in mitotic activity and cell size upon sympathetic stimulation (29,30). Experimentally, treatment with a β-adrenergic receptor agonist causes a profound weight-gain without being accompanied by increased secretory capacity (31).

Though parasympathomimetics are traditionally used in the clinic to stimulate the glands, experiments show that their excitation of the muscarinic receptors does not restore the marked reduction in gland size of the parasympathetically denervated gland, neither do parasympatholytics (like atropine ) mimic the weight fall caused by the denervation, findings showing that the gland size is not regulated by muscarinic receptors, but rather by parasympathetic neuropeptide-transmitters and (or) neuro-trophic factors of which we know little about (32).

Since the functional regeneration of the glands is likely to depend on an interplay between gland tissue and nerves, it is of interest to note, as judged from other experimental models, that electrical nerve stimulation promote the repair of damaged nerves (33,34).

## Conclusions

Since the specific factors controlling gland size and glandular activities are far from fully understood, comprehensive nerve stimulation as achieved by lingual nerve electrostimulation seems to be an excellent way to reactivate and regenerate the glands, while at the same time avoiding adverse effects. Bilateral stimulation of the lingual nerve could enhance the effect even more.
